# Peer Review of “Mask Use to Curtail Influenza in a Post–COVID-19 World: Modeling Study”

**DOI:** 10.2196/37172

**Published:** 2022-05-27

**Keywords:** mask, protection, COVID-19, influenza, transmission, intervention, infectious disease, respiratory, simulation, model, prevalence, efficacy

*This is a peer-review report submitted for the paper “Mask Use to Curtail Influenza in a Post–COVID-19 World: Modeling Study.”*

## Round 1 Review

### General Comments

The manuscript [1] entitled “Masks in Post COVID-19 World: Better Alternative to Curtailing Influenza?” undertakes the question as to whether the use of masks should continue after the COVID-19 pandemic for influenza. The authors use a susceptible-exposed-infected-recovered model to evaluate mask parameters.

### Specific Comments

The variety of combinations of mask prevalence and mask efficacy are very helpful and give strength to the paper.

#### Major Comments

1. An additional introductory paragraph on the susceptible-exposed-infected-recovered model would strengthen the manuscript and open it up to a wider audience, as this topic is of interest to many.

2. An additional 1 to 3 paragraphs in the Discussion are needed, comparing this study to similar studies.

3. I suggest the authors use color-blind–friendly colors for the figures.

#### Minor Comments

4. This statement needs rewording: “vaccines of course only have to be administered once while face masks need to be worn continuously.” I suggest separating this away from the rest of the sentence and making it a cleaner statement.

5. The last sentence of the Discussion is a run-on sentence. Please fix.
